# Exploring the Value of sC5b‐9 in the Diagnosis of Haematopoietic Stem Cell Transplant‐Associated Thrombotic Microangiopathy in Children

**DOI:** 10.1111/jcmm.70906

**Published:** 2025-10-22

**Authors:** Linlin Luo, Hao Xiong, Zhi Chen, Zhuo Wang, Fang Tao, Yu Du, Li Yang, Ming sun, Shanshan Qi, Wei Wang

**Affiliations:** ^1^ Wuhan Children's Hospital (Wuhan Maternal and Child Healthcare Hospital), Tongji Medical College Huazhong University of Science & Technology Wuhan China

**Keywords:** children, haematopoietic stem cell transplantation, sC5b‐9, transplant‐associated thrombotic microangiopathy

## Abstract

This study aims to explore the association between plasma human terminal complement complex C5b‐9 (sC5b‐9) levels and the occurrence of haematopoietic stem cell transplantation‐associated thrombotic microangiopathy (TA‐TMA) in children. The study retrospectively analysed the data of 131 patients who underwent allogeneic haematopoietic stem cell transplantation (allo‐HSCT) at the Department of Haematology‐Oncology between May 2020 and July 2023. The clinical data included dynamic data on plasma sC5b‐9 concentrations and analysed the potential of pre‐ and post‐transplantation levels for the prediction and diagnosis of TA‐TMA after allo‐HSCT. The median age of the cohort was 5.9 [3.4, 10.8] years, and it comprised 73 (55.8%) male and 58 (44.2%) female patients. The primary diseases were haematologic malignancy in 57 cases (43.5%), aplastic anaemia in 39 cases (29.8%) and Mediterranean anaemia in 13 cases (9.9%). Out of the 131 patients, 21 (14.7%) developed TA‐TMA, and the median time of onset was 88 [59, 136] days after transplantation. The 1‐year overall survival rate was significantly higher in the non‐TA‐TMA group (92.7% ± 2.5%) than in the TA‐TMA group (47.6% ± 10.7%) (χ^2^ = 35.71, *p* < 0.05). During TA‐TMA, sC5‐9 levels are significantly elevated (*p* < 0.05). However, the baseline levels of sC5b‐9 before allo‐HSCT were not related to the development of TA‐TMA after the procedure. The plasma sC5b‐9 concentration after allo‐HSCT in children is closely related to the occurrence of TA‐TMA, and 306.4 ng/mL is an optimal clinical cut‐off level above which TA‐TMA is indicated. This cut‐off level was associated with a sensitivity of 90.5% and a specificity of 86.6%. Nonetheless, the diagnosis of TA‐TMA should be established in conjunction with clinical manifestations and corroborated by additional laboratory test results.

## 1 Introduction

Allogeneic haematopoietic stem cell transplantation (allo‐HSCT) is an effective treatment for childhood haematologic malignancies, bone marrow failure syndromes, inherited metabolic diseases and primary immunodeficiency diseases. Transplantation‐related complications pose a serious threat to the quality of life and survival of patients. In addition to improvements in transplantation techniques, detecting and managing these complications on time is also an important strategy to combat their adverse effects on treatment outcomes and patient survival.

Transplant‐associated thrombotic microangiopathies (TA‐TMA) are some of the serious complications after allo‐HSCT characterised by microvascular thrombosis, thrombocytopenia, microangiopathic haemolytic anaemia and organ failure [1]. Its early clinical manifestations are atypical and include ineffective platelet transfusion, progressive haemoglobin decline, red blood cell fragments in peripheral blood, elevated lactate dehydrogenase levels, acute renal impairment, proteinuria, hypertension and so on [2]. TA‐TMA causes ecchymosis, petechial bleeding, renal impairment and neurological abnormalities and is associated with a mortality rate of nearly 65% [3, 4]. Correctly identifying TA‐TMA in the early stages through objective laboratory test indicators remains a challenging aspect of clinical treatment. Abnormal activation of the complement pathway plays a key role in the pathogenesis of TA‐TMA, and the soluble terminal complement complex (C5b‐9) is the common terminal effector of all complement pathways. Accordingly, Jodele et al. suggested that the expression level of sC5b‐9 should be used as a reference index for the diagnosis of TA‐TMA [5, 6]. The Chinese Expert Consensus on the Diagnosis and Treatment of Thrombotic Microangiopathy Associated with Haematopoietic Stem Cell Transplantation (2021) also lists elevated sC5b‐9 levels as one of the diagnostic criteria for TA‐TMA [7]. However, the cut‐off value of sC5b‐9 for the detection of TA‐TMA in post‐HSCT populations is unclear. In view of the lack of paediatric studies, this study aimed to explore the cut‐off value of plasma sC5b‐9 for the diagnosis of TA‐TMA in children by retrospectively analysing the clinical data of children who underwent allo‐HSCT at our centre.

## 2 Patients and Methods

### 2.1 Patient Population, Sample and Data Collection

**FIGURE 1:**
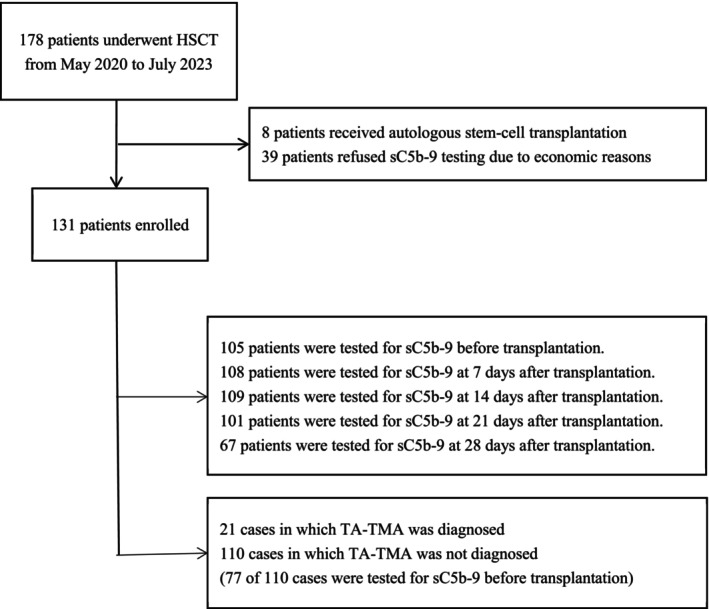
Study flow chart depicting the selection process and time points of sC5b‐9 testing.

This study included patients who underwent HSCT at the Wuhan Children's Hospital (Wuhan Maternal and Child Healthcare Hospital), Tongji Medical College, Huazhong University of Science and Technology, between May 2020 and July 2023. From an initial group of 178 patients, 47 were excluded either because they had undergone autologous stem‐cell transplantation (*n* = 8) or because they had refused sC5b‐9 testing (*n* = 39). A flow chart depicting the patient selection process is presented in Figure 1.

### 2.2 Diagnostic Criteria for TA‐TMA

The 131 patients who were finally included were divided into the TA‐TMA group (*n* = 21) or non‐TA‐TMA group (*n* = 110), with TA‐TMA diagnosed according to the criteria of the Chinese Expert Consensus on the Diagnosis and Treatment of Thrombotic Microangiopathy Associated with Haematopoietic Stem Cell Transplantation (2021) [7]. The Consensus has adopted the Jodele criteria for TMA diagnosis [2]. The diagnosis was confirmed on the basis of clear microvascular thrombosis changes on pathological examination or the presence of any five of the following seven criteria: (1) thrombocytopenia, (2) decrease in haemoglobin levels, (3) hypertension, (4) elevated lactate dehydrogenase levels, (5) proteinuria, (6) fragmented red blood cells in peripheral blood and (7) increased levels of sC5b‐9. All patients had to meet five of the seven criteria to make a diagnosis of TA‐TMA. Effective treatment is indicated by effective blood transfusion, increased platelet count, improved anaemia, decreased lactate dehydrogenase, disappearance of proteinuria, resolution of petechiae and ecchymosis and blood pressure within the normal range [8].

### 2.3 Detection of Plasma sC5b‐9 Concentration

Blood samples from all patients were sent for testing to a third‐party laboratory company. Peripheral blood from patients anticoagulated with EDTA was collected. The concentration of human terminal complement complex sC5b‐9 in plasma was detected using the sC5b‐9 enzyme‐linked immunosorbent assay (ELISA) kit, in conjunction with the ELISA reader EXL‐800TS, both provided by Bio‐Tek Instruments Inc., USA. We collected the patient's sC5b‐9 samples at pretransplant baseline, Days 7, 14, 21 and 28 after transplantation, as well as at the time of diagnosis for TA‐TMA patients.

### 2.4 Follow‐Up

The electronic medical records of the patients available at the hospital were reviewed, and follow‐up data were obtained through telephone interviews and clinic visits after discharge. Patients were followed up until 30 June 2024. The median follow‐up time was 23.7 [15.6, 32.4] months. Overall survival (OS) was defined as the time from the 1st day of HSCT to death or the last follow‐up.

### 2.5 Statistical Analysis

Statistical analysis was performed using SPSS 24. Nonnormally distributed data are presented as the median and interquartile range (M [Q1, Q3]), and comparisons between groups were performed using the Mann–Whitney *U* rank‐sum test. Kaplan–Meier survival analysis was conducted for univariate analysis of the survival rate. We utilised Receiver Operating Characteristic (ROC) curve analysis to assess the diagnostic effectiveness of sC5b‐9 in the context of TA‐TMA. The ROC curve provides a visual representation of a test indicator or model's performance by plotting the true positive rate (sensitivity) against the false positive rate (1—specificity) across various threshold levels. The cut‐off values were determined by the greatest Youden index (Youden index = sensitivity + specificity −1). The area under the curve (AUC) serves as a quantitative measure of the ROC curve's overall effectiveness. An AUC value approaching 1 signifies high diagnostic efficacy, whereas an AUC value of 0.5 denotes an absence of diagnostic utility. *p* < 0.05 was considered to indicate statistically significant differences. Data were graphed using GraphPad Prism 8 and SPSS 24.

### 2.6 Ethical Considerations

This study was reviewed and approved by the Wuhan Children's Hospital Ethics Committee (approval no. 2022R017‐E01).

## 3 Results

### 3.1 Demographic and Clinical Characteristics

The median age of the 131 included patients was 5.9 [3.4, 10.8] years, and the cohort comprised 73 (55.8%) male and 58 (44.2%) female patients. The primary diseases were haematologic malignancy in 57 cases (43.5%), aplastic anaemia in 39 cases (29.8%), Mediterranean anaemia in 13 cases (9.9%), haemophagocytic syndrome in 8 cases (6.1%), primary immunodeficiency diseases in 8 cases (6.1%), myelodysplastic syndrome in 4 cases (3.1%), Glanzmann thrombasthenia in 1 case (0.8%) and chronic active EBV infection in 1 case (0.8%). The transplantation methods included 81 (61.8%) haploidentical transplants, 26 (19.8%) HLA‐matched unrelated transplants, 13 (9.9%) HLA‐matched sibling transplants and 11 (8.4%) HLA‐mismatched unrelated transplants. With the exception of one patient who received cord blood transplantation, the remaining 130 patients underwent peripheral blood stem cell transplantation. Haematopoietic reconstitution was achieved in all 131 cases. The median time to engraftment was 15 days for both neutrophils and platelets. The 100‐day, 2‐year and 3‐year OS rates after HSCT were 94.6% ± 2.0%, 85.4% ± 3.1% and 83.8% ± 3.2%, respectively.

### 3.2 Clinical Data of Patients With TA‐TMA

**FIGURE 2:**
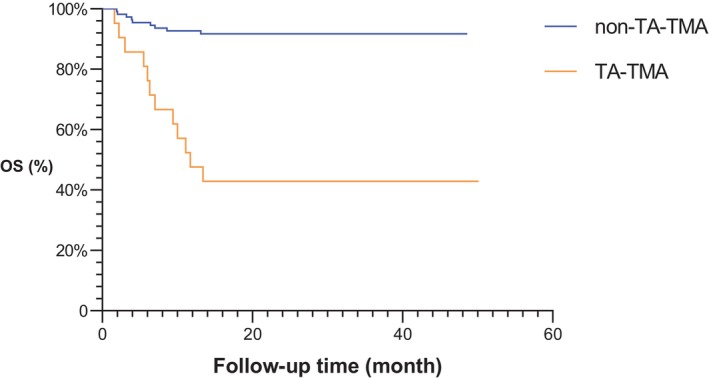
Overall survival rate in the TA‐TMA group and non‐TA‐TMA group.

A total of 21 patients (14.7%) were diagnosed with TA‐TMA in the study sample, out of whom three experienced a second episode of TA‐TMA. The median time of onset of TA‐TMA was 88 [59, 136] days. Children without TA‐TMA were considered as the comparison group (*n* = 110) for analysis. There was no significant difference in gender, primary disease, ABO compatibility, GVHD prophylaxis, stem cell population (CD34 + or nucleated cells) or time required for neutrophil and platelet engraftment between the two groups (Table 1). We evaluated the sC5b‐9 levels in patients with TA‐TMA during the disease and in those without TA‐TMA before transplantation as a reference point. In the univariate analysis, there were statistically significant differences between the TA‐TMA group and the non‐TA‐TMA group in terms of age (*p* = 0.00) and sC5b‐9 (*p* = 0.00). Subsequent logistic regression analysis demonstrated that both age (OR = 1.158, 95% CI 1.009–1.328, *p* = 0.037) and sC5b‐9 levels (OR = 1.009, 95% CI 1.008–1.013, *p* < 0.01) were significantly associated with the incidence of TA‐TMA, identifying them as independent risk factors. In the subsequent Cox proportional hazards model, sC5b‐9 remained a significant predictor (HR = 1.003, 95% CI 1.001–1.005, *p* < 0.01), while the effect of age was no longer significant (*p* = 0.27). The 1‐year OS rate after transplantation was significantly lower in the TA‐TMA group (47.6% ± 10.7%) than in the non‐TA‐TMA group (92.7% ± 2.5%) (χ^2^ = 35.22, *p* < 0.05). The mortality rate of children in the TA‐TMA group was significantly higher than that in the non‐TA‐TMA group (χ^2^ = 35.71, *p* < 0.05) (Figure 2). By the end of the follow‐up period, eight of the children with TA‐TMA had died from the condition, resulting in a mortality rate of 38.1% associated with TA‐TMA. Furthermore, three children succumbed to sepsis, and one child died due to the primary disease, resulting in an overall mortality rate of 57.1%.

**TABLE 1:** Demographic and clinical characteristics of the study population (*N* = 131).

	TA‐TMA (*n* = 21)	Non‐TA‐TMA (*n* = 110)	*p*
**Age, median (IQR)**	12.1 (5.7, 14.3)	5.6 (3.0, 9.0)	0.00
**Sex**			0.46
Male	10 (47.6%)	62 (56.4%)	
Female	11 (52.4%)	48 (43.6%)	
**Primary disease**			0.40
Malignant disease	8 (38.1%)	53 (48.2%)	
Nonmalignant disease	13 (61.9%)	57 (51.8%)	
**Donor type**			0.14
Sibling	16 (76.2%)	65 (59.1%)	
Others	5 (23.8%)	45 (40.9%)	
**Donor/recipient blood type**			0.56
ABO compatible	10 (47.6%)	60 (54.5%)	
ABO incompatible	11 (52.4%)	50 (45.5%)	
**GVHD prophylaxis**			
PTCy administered	8 (38.1%)	43 (40.6%)	0.83
PTCy not administered	13 (61.9%)	63 (59.4%)	
**sc5b‐9**	416 (356, 649.4)	190 (151, 9263.7)	0.00
**Mononuclear cell count (×10** ^**8**^ **/kg)**	8.4 (7.7, 26.6)	8.8 (7.8, 17.9)	0.36
**CD34+ cell count (×10** ^**6**^ **/kg)**	8.0 (4.2, 14.1)	6.6 (4.6, 10.6)	0.49
**Time to neutrophil engraftment, days**	15.0 (12.3, 16.0)	14.0 (12.0, 15.0)	0.14
**Time to platelet engraftment, days**	15.5 (12.0, 20.0)	15.0 (12.0, 19.0)	0.92

### 3.3 The Significance of sC5b‐9 Levels in the Diagnosis of TA‐TMA

**FIGURE 3:**
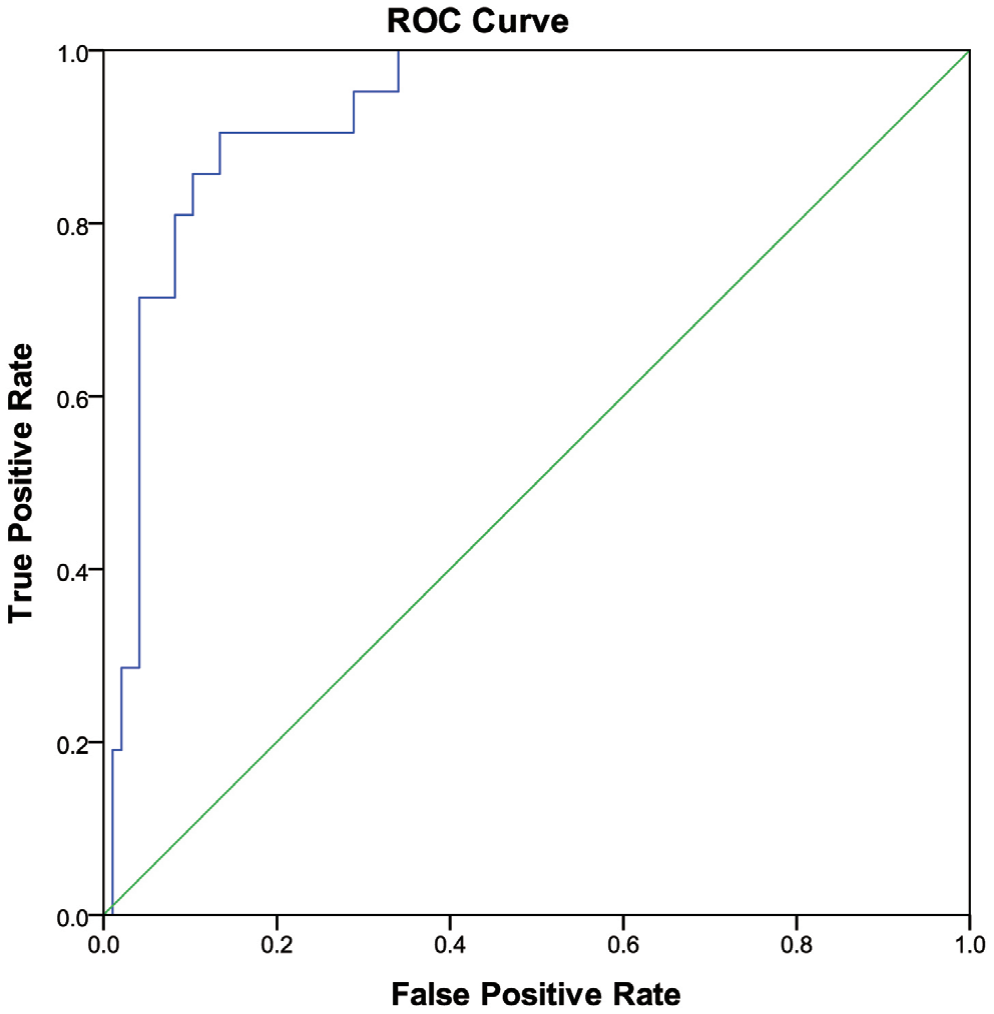
ROC curve showing the sensitivity and specificity of sC5b‐9 at a cut‐off value of 306.4 ng/ml.

In the preceding text, our analysis utilising binary logistic regression revealed a statistically significant association between sC5b‐9 levels and the onset of TA‐TMA (*p* < 0.05). Consequently, we constructed a receiver operating characteristic (ROC) curve using pretransplantation sC5b‐9 data from individuals without the disease (*n* = 97) alongside data from patients diagnosed with TA‐TMA during the disease course (*n* = 21). The analysis of the ROC curve demonstrated that the AUC value for sC5b‐9 was 0.929, with a 95% confidence interval ranging from 0.879 to 0.980. This suggests a high diagnostic efficacy for TA‐TMA. Notably, when the optimal threshold was established at 306.4 ng/mL, the sensitivity and specificity of this biomarker were 90.5% and 86.6%, respectively (Youden index = 0.53). These findings indicate that at this threshold, sC5b‐9 effectively differentiates between patients with TA‐TMA and those without the condition (Figure 3). We divided the patients into a high sC5b‐9 group and a low sC5b‐9 group based on whether sC5b‐9 was greater than 306.4 ng/mL. There was no significant difference in gender, age, primary disease, ABO compatibility, GVHD prophylaxis, stem cell population (CD34 + or nucleated cells) or time required for neutrophil and platelet engraftment between the two groups (Table 2). The incidence of TMA was significantly higher in the high sC5b‐9 group compared to the low sC5b‐9 group (*p* < 0.01). Logistic regression analysis showed that high sC5b‐9 levels were associated with an increased risk of TA‐TMA (OR = 72.580, 95% CI 7.88–668.337, *p* < 0.01).

**TABLE 2:** Comparison of characteristics between high and low sC5b‐9 groups.

	High sC5b‐9 group (*n* = 32)	Low sC5b‐9 group (*n* = 86)	P
**Age, Y(IQR)**	7.7 (3.4, 13.7)	5.9 (3.5, 10.0)	0.17
**Gender**			0.16
Male	14 (43.8%)	50 (58.1%)	
Female	18 (56.2%)	36 (42.9%)	
**Primary disease**			0.13
Malignant disease	11 (34.4%)	43 (50.0%)	
Nonmalignant disease	21 (65.6%)	43 (50.0%)	
**Donor type**			0.20
Sibling	22 (68.8%)	48 (55.8%)	
Others	10 (31.2%)	38 (44.2%)	
**Donor/recipient blood type**			0.79
ABO compatible	17 (53.1%)	48 (55.8%)	
ABO incompatible	15 (46.1%)	38 (44.2%)	
**TA‐TMA**			0.00
Yes	19 (59.4%)	2 (2.3%)	
No	13 (40.6%)	84 (97.7%)	
**GVHD prophylaxis**			0.69
PTCy	11 (34.4%)	33 (38.4%)	
Non‐PTCY	21 (65.6%)	53 (61.6%)	
**MNC (X10^8/KG)**	8.9 (7.7,17.2)	8.2 (7.6,17.7)	0.23
**CD34+ cells (X10^6/KG)**	7.1 (4.1,13.1)	6.6 (4.6,10.3)	0.75
**Neutrophil engraftment**	15 (13,16)	14 (12,15)	0.06
**Platelet engraftment**	17 (13,21)	14 (12,19)	0.36

### 3.4 The Significance of sC5b‐9 Levels in Predicting the Occurrence of TA‐TMA

**FIGURE 4:**
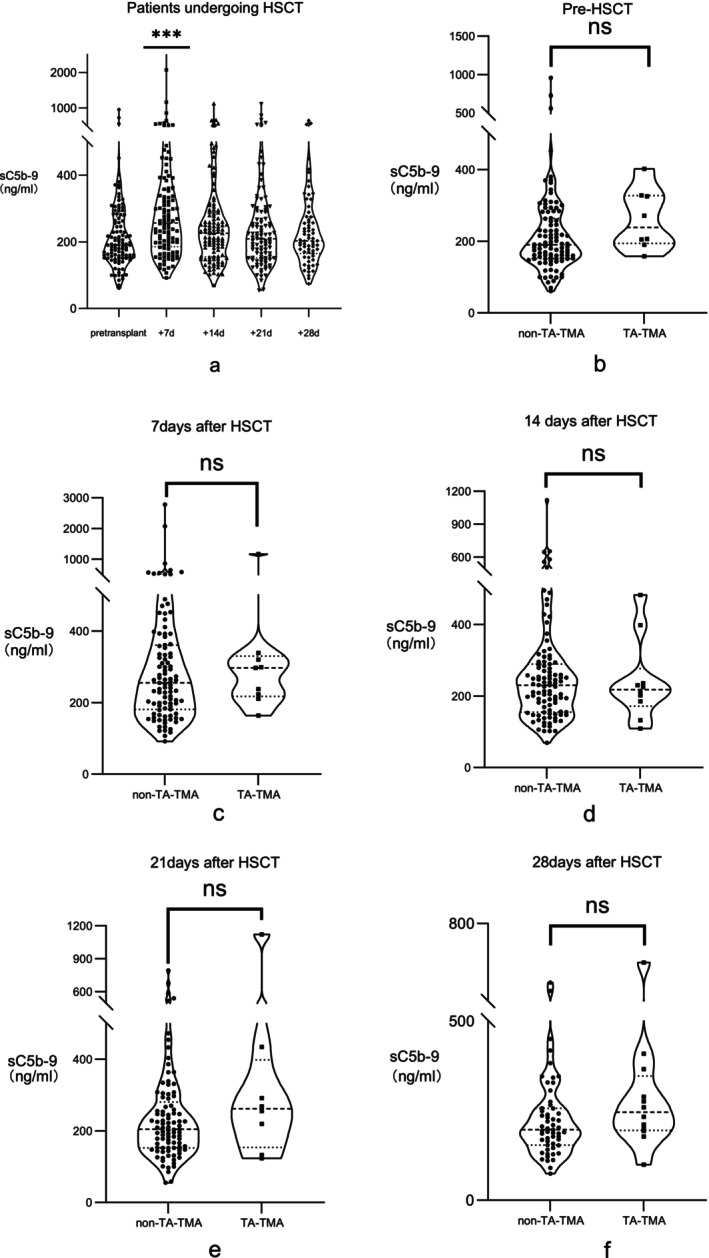
Pre and post‐transplantation levels of sC5b‐9. (a) The levels of sC5b‐9 at 7 days after transplantation were significantly higher than those before transplantation and at the other time points after transplantation. (b–f) The pretransplantation sC5b‐9 levels were not significantly different between patients with and without TA‐TMA.

The median plasma levels of sC5b‐9 before HSCT and at 7, 14, 21 and 28 days after HSCT were 90.6 [154.1–276.5], 256.6 [185.7–356.4], 229.6 [155.5–280.0], 209.0 [151.8–290.5] and 202.6 [164.8–275.0] ng/ml, respectively (Figure 4). The results showed that the levels of sC5b‐9 at 7 days after transplantation were significantly higher than those before transplantation and at the other time points after transplantation (χ^2^ = 18.6, *p* < 0.05). The pretransplantation sC5b‐9 levels were not significantly different between patients with and without TA‐TMA (*p* = 0.09). Concurrently, we assessed the alterations in sC5b‐9 levels from baseline prior to transplantation to 28 days post‐transplantation. Our analysis revealed that the variations in these changes between the TA‐TMA group and the non‐TA‐TMA group did not reach statistical significance (*p* = 0.09). This indicates that the baseline levels of sC5b‐9 before allo‐HSCT were not associated with the onset of TA‐TMA after transplantation.

### 3.5 The Significance of sC5b‐9 Levels in Predicting the Therapeutic Efficacy of TA‐TMA

During the treatment process, each episode of illness in patients was managed using a standardised regimen that comprised the infusion of fresh frozen plasma, administration of low‐molecular‐weight heparin and dose‐adjusted calcineurin inhibitors as the foundational therapeutic approach. In this study, 15 patients underwent therapeutic plasma exchange, while 18 patients received rituximab therapy, with 11 patients potentially receiving both treatments. Due to economic constraints and limited access to medications, only two patients were treated with defibrotide, and a mere two patients received eculizumab. Among the patients treated with eculizumab, one patient survived, while the other patient died due to severe infections. Patients were stratified into two cohorts according to therapeutic efficacy: an effective treatment group and an ineffective treatment group. At the time of diagnosis, the serum sC5b‐9 concentration was 420 [404.7, 774.9] ng/mL in the ineffective treatment cohort and 416 [307, 648] ng/mL in the effective treatment cohort. The difference in sC5b‐9 levels between the two groups was not statistically significant (*p* = 0.18).

## 4 Discussion

The present study evaluates the potential of sC5b‐9 as a predictive marker of post‐transplantation TA‐TMA in paediatric patients who have undergone allo‐HSCT. The findings indicate that sC5b‐9 can predict TA‐TMA with high sensitivity and specificity when a cutoff value of 306.4 ng/mL is applied. This finding could have important implications in the clinical context, as the cutoff value for this group of patients has not been clarified prior to this. In recent years, the range of mortality associated with TA‐TMA has decreased from 0.5%–76% to 8.2%–39% [9, 10]. The incidence of TA‐TMA and the mortality rate of transplant patients at our centre were 11.8% and 57.1%, respectively, which is similar to previous reports [10]. With regard to the predictive potential of sC5b‐9, Jodele initially found that the concentration of sC5b‐9 in TA‐TMA patients increased at the time of disease onset [5]. Furthermore, Horváth reported that an increase in sC5b‐9 during the 28 days after transplantation can predict TA‐TMA in later stages [11]. In contrast to their observation, in the current study, the level of sC5b‐9 in the TA‐TMA group at 28 days after transplantation was higher than that in the non‐TA‐TMA group, but the difference was not significant. Mezö proposed that a 66 ng/mL increase in plasma sC5b‐9 levels from baseline at 28 days post‐transplantation could predict the development of TA‐TMA [12]. Additionally, this study found that the change in sC5b‐9 levels from before to 28 days after transplantation could not effectively predict the occurrence of TA‐TMA, which is inconsistent with the reports by Mezö et al. [12]. Qi et al. suggested that plasma sC5b‐9 levels were significantly elevated in patients with TA‐TMA [13], and Jodele et al. found that a doubling of sC5b‐9 levels from baseline at HSCT, even within normal limits, raises the risk of organ injury in TA‐TMA [14]. Furthermore, Koo et al. reported that an increase in sC5b‐9 levels from pre‐HSCT to Day 7 correlates with a greater risk of developing TA‐TMA [15]. Additionally, this study has investigated the correlation between the elevation of sC5b‐9 levels in serum at baseline before HSCT and on Days 7, 14, 21 and 28, and the risk of TA‐TMA occurrence. Regrettably, no significant correlation was identified between these two factors. This limitation may be attributed to the relatively small sample size employed in our study. We anticipate that future research with a larger sample size will provide validation for our findings.

Although sC5b‐9 is believed to have diagnostic and prognostic value in TA‐TMA, there is no established cutoff value for diagnosis. Further, in a retrospective study, the median concentration of sC5b‐9 in 20 adults with TA‐TMA was determined as 394 (300–519) ng/ml [13]. Jodele et al. reported that in a study involving children and adolescents with TA‐TMA, the median plasma sC5b‐9 concentration was significantly elevated to 498.2 ng/mL in patients who succumbed to the condition. This finding underscores the potential prognostic value of this biomarker in identifying high‐risk cases [5]. Our data show that a sC5b‐9 concentration of 306.4 ng/mL can be used as the optimal cut‐off point. This cut‐off value had a sensitivity and specificity of 94.1% and 89.8%, respectively, for the diagnosis of TA‐TMA. However, it is important to note that reference ranges for sC5b‐9 can vary across different laboratories [14, 15]. In our study, we found that the sC5b‐9 increases 7 days after transplantation, which may be related to the early immune response and inflammatory processes following transplantation. Therefore, our conclusions may not be applicable to the diagnosis of TA‐TMA around 1 week after transplantation. Meanwhile, the above conclusion also awaits further validation by future prospective studies.

Further, the treatment‐ineffective TA‐TMA group had slightly higher sC5b‐9 levels than the treatment‐responsive group in our study, although the difference was not significant. In a previous study, higher sC5b‐9 concentrations were observed in the deceased group of TA‐TMA patients who did not receive treatment in comparison with the group that survived [12]. According to Jodele et al. [16], patients exhibiting pretreatment sC5b‐9 levels at least twice the normal threshold (> 488 ng/mL) necessitate a sustained eculizumab serum concentration of 99 mg/mL for a duration of 11–13 days to achieve normalisation of sC5b‐9. In contrast, patients with pretreatment sC5b‐9 levels below double the normal value (< 488 ng/mL) require only 2–5 days for normalisation of sC5b‐9. Simultaneously, a shorter duration of elevated sC5b‐9 levels correlates with improved clinical outcomes. In our study, two patients underwent eculizumab treatment. Unfortunately, due to existing constraints, we are currently unable to monitor the concentration of eculizumab. In the future, we aim to collaborate with other laboratories to conduct pertinent research, with the objective of enhancing treatment for patients with TA‐TMA. Jodele et al. found in another study that monitoring the changes in sC5b‐9 levels in TA‐TMA patients is equally important. Among patients who did not receive targeted therapy, those who succumbed exhibited a prolonged duration of elevated sC5b‐9 levels compared to survivors (23 vs. 14 days) [14]. Furthermore, we intend to conduct prospective studies to further validate the prognostic significance of sC5b‐9 in the assessment of TA‐TMA. The sample size of this study is relatively limited, necessitating validation of our findings through future large‐scale, multicentre prospective trials.

In summary, there was a positive correlation between plasma sC5b‐9 concentrations and TA‐TMA in children who underwent allo‐HSCT, with sC5b‐9 levels of ≥ 306.4 ng/mL indicating high diagnostic sensitivity and specificity for TA‐TMA. Thus, monitoring the concentration of sC5b‐9 after transplantation can aid in the diagnosis of TA‐TMA in children. However, its prognostic potential in this group of patients requires further investigation in large‐scale multicentre patient populations.

## Author Contributions

**Linlin Luo:** conceptualization (equal), data curation (equal), methodology (equal), writing – original draft (equal). **Hao Xiong:** conceptualization (equal), methodology (equal), writing – review and editing (equal). **Zhi Chen:** data curation (equal), formal analysis (equal), investigation (equal). **Zhuo Wang:** data curation (equal), formal analysis (equal), investigation (equal). **Fang Tao:** data curation (equal), formal analysis (equal), investigation (equal). **Yu Du:** data curation (equal), formal analysis (equal), validation (equal). **Li Yang:** formal analysis (equal), investigation (equal), validation (equal). **Ming sun:** data curation (equal), investigation (equal), methodology (equal), validation (equal). **Shanshan Qi:** methodology (equal), validation (equal), writing – original draft (equal). **Wei Wang:** methodology (equal), validation (equal).

## Ethics Statement

Ethical approval was obtained from the Ethics Committee of Wuhan Children's Hospital (2022R017‐E01).

## Consent

The study was done after agreement from the local ethics committee, and written informed consent was obtained from all participants and/or their guardians.

## Conflicts of Interest

The authors declare no conflicts of interest.

## Data Availability

The data that support the findings of this study are available on request from the corresponding author. The data are not publicly available due to privacy or ethical restrictions.
